# Detectable SARS-CoV-2 specific immune responses in recovered unvaccinated individuals 250 days post wild type infection

**DOI:** 10.1371/journal.pone.0325923

**Published:** 2025-06-11

**Authors:** Nikolas Weigl, Claire Pleimelding, Leonard Gilberg, Duc Huynh, Isabel Brand, Jan Bruger, Jonathan Frese, Tabea M. Eser, Mohamed I. M. Ahmed, Jessica M. Guggenbuehl-Noller, Renate Stirner, Michael Hoelscher, Michael Pritsch, Christof Geldmacher, Sebastian Kobold, Julia Roider, on behalf of the KoCo19-/ORCHESTRA-study group

**Affiliations:** 1 Division of Clinical Pharmacology, Department of Medicine IV, LMU University Hospital, LMU Munich, Munich, Germany, Member of the German Center for Lung Research (DZL); 2 Department of Infectious Diseases, LMU University Hospital, LMU Munich, Munich, Germany; 3 Institute of Infectious Diseases and Tropical Medicine, LMU University Hospital, LMU Munich, Munich, Germany; 4 Fraunhofer Institute for Translational Medicine and Pharmacology ITMP, Immunology, Infection and Pandemic Research, Munich, Germany; 5 German Center for Infection Research (DZIF), Partner Site Munich, Munich, Germany; 6 German Cancer Consortium (DKTK), Partner Site Munich, a partnership between the DKFZ Heidelberg and the LMU University Hospital, Munich, Germany; 7 Helmholtz Zentrum München, Research Center for Environmental Health (HMGU), Neuherberg, Germany; 8 Einheit für Klinische Pharmakologie (EKLiP), Helmholtz Zentrum München—German Research Center for Environmental Health, Munich, Germany; University of the Witwatersrand, SOUTH AFRICA

## Abstract

Memory T cells play an important role in mediating long-lasting adaptive immune responses to viral infections, such as SARS-CoV-2. In the context of the latter, much of our current knowledge stems from studies in vaccinated individuals or repeatedly infected individuals. However, limited knowledge is available on these responses in fully naive individuals in German communities. We performed immunophenotyping of a previously naive SARS-CoV-2 cohort in convalescent individuals after asymptomatic to moderate COVID-19. The samples were collected median 250 days post infection during the first wave of the COVID pandemic in Germany (March – May 2020). In this cohort of 174 individuals, we phenotyped different leukocyte cell populations in peripheral blood (B, T and Natural Killer cells). We then assessed the serostatus against the SARS-CoV-2 antigens Nucleocapsid (N) and Spike subunit (S1) with its receptor binding domain (RBD), as these are important correlates of protection, by testing for presence of immunoglobulin G (IgG) antibodies. We also measured IgG antibody responses against the N antigen of the common cold coronaviruses HCoV-OC43, HCoV-HKU1, HCoV-NL63 and HCoV-229E, to determine possible cross-reactivity. In a subset of the cohort (n = 76), we performed intracellular staining assays (ICS) after stimulation with SARS-CoV-2 and HCoV antigens. Key findings are significant differences in frequency of CD4+ memory T cell populations, notably CD4+ T_EM_ and CD4+ T_EMRA_ cells, between the group of SARS-CoV-2 positive individuals and the control group. These differences correlated with cytokine production (TNFα, IFNγ) after stimulation with SARS-CoV-2 peptides, indicating a specific T cell immune response. In conclusion, a clear memory T cell and humoral response can be detected up to 250 days post mild to moderate COVID-19 disease. Our results underline findings reported by others indicating a lasting cellular immune response even in a population which previously had not been exposed to SARS-CoV-2.

## Introduction

Since 2019, the then newly emerged severe acute respiratory syndrome coronavirus 2 (SARS-CoV-2) [1] and the following coronavirus disease 2019 (COVID-19) pandemic have had a significant impact on daily life around the globe. Substantial research efforts have covered numerous aspects of the virus and its global spread. Research into generating and maintaining a long-lasting immune response through infection and/or vaccination was and still is playing an important role in understanding the COVID-19 pandemic [2,3]. In our study, we engaged in understanding the immunological processes towards the wild-type virus in a SARS-CoV-2 naive population during the ‘pre-vaccination-period’. Nevertheless, this knowledge is of crucial importance to handle our current situation, where a globally endemic virus [4,5] and its new sub-lineages (i.e., beta, gamma, delta) with high evolving potential [6] are an ongoing challenge to the global population.

SARS-CoV-2 infection shows itself in a broad variety of clinical manifestations: While asymptomatic infections regularly occur, symptomatic infections mostly result in a mild to moderate disease. This includes flu-like symptoms such as fever, fatigue and dry cough [7]. Severe clinical courses of COVID-19 can lead to respiratory failure and death, mostly reported in the elderly or persons with underlying health conditions and immunodeficiency [7,8]. Demographic factors including sex, ethnicity and socioeconomic status have also been discussed as contributing to disease outcome [9,10].

Recent findings concerning humoral and cellular immunity to SARS-CoV-2 provide insights into effectiveness of vaccines [11,12] and longevity of immune responses of convalescent individuals [13,14]. Others analysed possible cross-reactivity between variants or other coronaviruses [15,16]. The endemic human coronaviruses (HCoVs) categorized in alpha-coronaviruses NL63, 229E and beta-coronaviruses HKU1 and OC43 (and now SARS-CoV-2) [6], have been investigated for their potential to mediate protection from severe SARS-CoV-2 infection via cross-reactive immune responses [17–19]. All coronaviruses share peptide sequence homologies between important immunological proteins including the spike (S) surface glycoprotein or the nucleocapsid (N) and membrane (M) proteins. The C-terminal epitopes in the spike protein show the highest homology between the coronaviruses [20,21]. The homology serves as an important factor in generating cross-reactive antibodies [22]. Furthermore, these antigens can evoke T cell dominated responses, defined by two important types of cluster of differentiation molecules (CD4+ and CD8+) [23–25]. A mapping of cross-reactive T cell epitopes across the SARS-CoV-2 genome demonstrated a range of reactive, pre-existing memory CD4+ cells in unexposed individuals. Most of these epitopes are derived from the spike antigen, especially the C-terminal epitopes, as mentioned above [20,24,25].

Neutralizing antibodies were widely acknowledged as the main correlate of protection from SARS-CoV-2 infection, especially during the early phase of the pandemic [26,27]. Nevertheless, the importance of T cells mediating long-lived and cross-reactive protection from COVID-19 has been recognized in the face of ever-changing viral variants [28,29]. This includes helper T cells (T_H_) expressing CD4-phenotypes coordinating adaptive immunity through synergy of various cytokines, as well as CD8+ cytotoxic-T (T_C_) cells necessary for the elimination of virus-infected cells [30,31].

In this project, we performed immunophenotyping including antigen-specific cytokine-release, memory T cell and serological responses against SARS-CoV-2 and the HCoVs 229E, HKU1, NL63 and OC43. Notably, our cohort consists of convalescent individuals after their first SARS-CoV-2 infection during the first wave of the COVID-19 pandemic between March and May 2020 in Germany [32] and contributes to existing community research on SARS-CoV-2 in other countries [33]. In this timespan, the wild-type variant was dominant, preceding the B.1.1.7 (alpha) variant of concern (VOC) [34]. The goal of the study was to determine long-lasting immunological impact after SARS-CoV-2-infection as well as potential correlations to pre-existing endemic coronavirus immunity.

## Materials and methods

### Study design and cohort

In this study, we performed serological and cellular immunoassays on blood samples collected in 2020 and early 2021 after the first wave of SARS-CoV-2 infections. The subjects were part of a previously described cohort that included SARS-CoV-2 positive tested individuals and their household members, located exclusively in the city of Munich, Germany [35].

Individuals who had tested PCR positive during March and April 2020 were initially contacted by the local Health Department and invited to participate in COVID-19 related studies. In total 216 households (567 participants) that showed interest were included between 29^th^ September 2020 and 27^th^ January 2021 by either house visit or at the study center, at the Institute of Infectious Diseases and Tropical Medicine, University Hospital, LMU Munich. To investigate immunological response, participants were asked to provide a venous blood sample. All participants gave their written consent before the blood sample was taken or the questionnaire completed. During the first visit, they received an information sheet and were verbally informed about all the important aspects of the study. After clarifying any questions with the study team, the participants signed their consent. They were also given the option of refusing some aspects of the study and not having to provide all samples (venous blood, capillary blood, sputum, nasopharyngeal swab).

All minors between the ages of 8 and 17 who were willing to participate in the study had to obtain the consent of their parents or legal guardians. Adolescents between the ages of 13 and 17 were allowed to have a larger blood sample taken with their consent. Only a capillary blood sample was taken from children between the ages of 8 and 13.

Symptom and household related data was collected via the mobile data collection tool OpenDataKit (ODK) [36].

As a SARS-CoV-2 negative control group, we recruited 36 households (98 participants) from the related KoCo-19 study. Members of these households had tested negative for SARS-CoV-2 specific antibodies multiple times and did not report any COVID-19 related symptoms during the first year of the pandemic [35,37,38]. We selected 15 households with 33 control subjects, which demographically matched our index and exposed group, for further experiments.

### PBMC and plasma isolation

For isolation of peripheral blood mononuclear cells (PBMCs), 30 mL CPDA (Citrate Phosphate Dextrose Adenine) blood in 3 x 9 ml (Vacuette, Art. Nr.: 455056) and 1 x 5 ml tubes (BD Vacutainer®, Na-Heparin (17 IU/ml), Art.Nr.: 143427) was centrifuged at 400 g for 10 minutes at room temperature. For each CPDA tube, 1 ml of plasma layer was collected and frozen in a −80 °C freezer.

The remainder of the blood mixture was divided equally into 2 falcon tubes of 50 mL and a Hanks Solution (HBSS Hanks solution, Sigma-Aldrich, Cat. No. H9394-500 ml +1% Glutamine (BioWhittaker, Lonza Cat. No.: BEBP17-605E) 200mM in 0,85% NaCl, +1% Penicillin/Streptomycin (Sigma-Aldrich Cat. No.: P433 - 100 ml), +1% Hepes (Sigma-Aldrich, Cat. No.: H0887 - 100 ml)) was added until the 32 ml mark. The blood suspension was slowly filled into falcon tubes with a 15 ml Ficoll-Paque (Sigma-Aldrich, Cat. No.: Histopaque-10771) and centrifuged at 1000 g for 20 minutes at room temperature.

PBMCs were harvested, washed with 50 mL Hanks solution in a falcon tube and again centrifuged at 520 g for 15 minutes at room temperature. Afterwards, the supernatant was discarded, and the cell pellets were re-suspended in 10 mL Hanks solution.

We used a CASY-1 (Schärfe System GmbH) for automated cell counting by adding 50µl of our Hanks cell pellet solution with 10 ml of Casyton (OLS OMNI Life Bioscience, Cat. No.: 5651808). The device can analyze the cells using electric current exclusion and pulse area analysis. Another 10 µl were pipetted into 10 µl of diluted Trypan Blue (1:4 in PBS) to check viability with a haemocytometer under our microscope (Leica, DM IL). We counted blue and white shining leukocytes in one haemocytometer cell, sorting out red erythrocytes. All blue dyed leukocytes were considered dead due to their inability to discharge the dye. Then the ratio of living cells and all cells (living and dead) was formed to obtain a viability percentage. After confirming viability and cell number, the cells were centrifuged again for 5 minutes, at 4°C, with 400g. Then the cells were diluted in a concentration of 10 million cells/ml (90% fetal calf serum (FCS), Sigma-Aldrich, Cat. No.: S0615-500ML + 10% DMSO, Roth, Cat: 4720.4) for cryopreservation in cryo-tubes (Nunc, Thermo Fisher Scientific, Cat. No.: 377267). The tubes were stored for one night in our −80° C freezer and then transferred in a liquid nitrogen tank.

All plasma and PBMC samples were isolated and frozen within 3.5 hours past their arrival and had a confirmed viability of >95% at the time of freezing.

### Serological testing

To determine the seroprevalence of antibodies against SARS-CoV-2 and the common cold coronaviruses (CCCs), we used the Line-immunoassay recomLine SARS-CoV-2 IgG from Mikrogen GmbH, Neuried, Germany. The immunostrips contained the N antigen of SARS-CoV-2, HCoVs 229E, NL63, OC43, HKU1, the receptor-binding domain antigen (RBD) of the SARS-CoV-2 spike protein and the S1 subunit antigen. The binding of immunoglobulin (IgG) antibodies was visualized by the apparition of dark strips, through binding of anti-human immunoglobulin-antibodies (IgG) with a horseradish-peroxidase reacting to the chromogenic substrate tetramethylbenzidine. The test was performed according to the manufacturer’s protocol (Art. Nr.: 7374, protocol: GARLCS002D_2020-08).

A reaction-control and antibody-control strip provided proof of the right execution of the test. Sensitivity (96.3%) and specificity (98.8%) are reported as provided by the manufacturer.

The conversion of the qualitative results of the serotest was performed by taking high resolution pictures of the test strips and subsequently measuring the strength of the answer using the RGB-value of the respective lines, similar to Dugas et al. [39], as a method to quantify immunological test strips [40,41]. The measurements of the RGB-values were performed with the software ImageJ [42].

### Intracellular cytokine staining assay (ICS)

For the intracellular staining assay (ICS), an amount of 20–30 million PBMCs per patient was used. PBMCs were washed in a 15 ml falcon tube with 10 mL of R10 (RPMI 1640 medium, Sigma-Aldrich, Cat.No.: R5886 + 10% FCS, + 1% Streptomycin/Penicillin, +1% vial L-Glutamine). Then, the PBMCs were centrifuged for 10 minutes at 1500 rpm at 4° C. The supernatant was discarded, the PBMCs re-suspended in 2 ml R10 solution and washed again as described above with a total of 10 ml R10. Centrifugation was then performed at room temperature. The supernatant was again discarded, a viability check and cell counting was performed as previously described. PBMCs were resuspended in R10 with a 1,5 million PBMCs per 200 µl ratio (7.5 million cells/mL).

The PBMCs were plated into a 96 U well plate, each well containing 200 µl and incubated for 2 hours at 37 °C, 5% CO_2_ humidified.

During the incubation period the peptide solution was prepared using PepTivator® SARS-CoV-2 (Miltenyi Biotec) for the Membrane (M) (Cat. No.: 130-126-703) and Nucleocapsid (N) (Cat. No.: 130-126-698) SARS-CoV-2 antigen and JPT PepMix SARS-CoV-2 for subunit 1 and 2 of the Spike Glycoprotein (S1 and S2) (JPT, Cat. No.: PM-WCPV-S-1/ -2), as well as the JPT PepMix antigens for the subunits 1 and 2 of the HCoVs 229E, OC43, NL63 and HKU1 (JPT Cat. Nrs.: PM-229E-S-1/-2, PM-OC43-S-1/-2, PM-NL63-S-1/-2, PM-HKU1-S-1/ -2) and a JPT PepMix positive control pool (CEF) (JPT Cat. No.: PM-CEF-S-1) with specific epitopes for cytomegalovirus, Epstein-Barr and Influenza virus. Each of the antigen kits was aliquoted beforehand according to the manufacturer’s guidelines with DMSO (0.2 µl = 1 µg/ml). Using the freely available blast website of the NIH [43] we could identify the homology between the peptide sequences used in the stimulation and the surface protein of the wild-type SARS-CoV-2 virus. We did find a homology percentage of 37.93% for HCoV OC43, 32.24% for 229E, 30.77% for NL63 and 35.82% for HKU1. For the Peptivator Nucleocapsid and Membrane Protein, the homology showed 100%.

Each aliquot was re-suspended in 1 ml R10 and 200 µl added to each well (0.2 µl peptide per well). 2 µl SEB solution (Staphylococcal Enterotoxin B in PBS, 1 µg/mL, Sigma-Aldrich Cat. No.: S4881-1MG) was used as a positive control. After an incubation period of 2 h at 37 °C, 5% CO2, 95% humidity, we performed a washing step and added 0.6 µl of Golgi Plug (Brefeldin A, BD Biosciences, Cat. No.: 554724) to every well. An additional dual-negative control only contained 0.2 µl of DMSO to mimic the DMSO the peptides were aliquoted in. After adding the Brefeldin A, the cells were incubated overnight for another 14 h hours (16 h peptide stimulation in total). After incubation, the PBMCs were centrifuged for 5 minutes at 400 g, washed with 200 µl FACS-Buffer (PBS +2% FCS) and then stained with the antibody master-mix for extracellular stimulation. The master-mix contained 0.5 µl of every antibody per well. CD3 (= Alexa Fluor 700 anti-human CD3, BioLegend, Clone HIT3a, Cat. No.: 300324), CD4 (= PE/Dazzle 594 anti-human CD4, Clone A16.1A1, Cat. No.: 357412), CD8 (= Brilliant Violet 510 anti-human CD8, BioLegend, Clone SK1, Cat. No.: 344732). Every well was then diluted in 100 µl FACS-buffer plus 1 µl TruStain (FcReceptorBlock, BioLegend, Cat. No.: 422302) and 0.1 µl Life-Dead-solution (invitrogen eBioScience Fixable Viability Dye eFluor780, Cat. No.: 65-0865-14). After an incubation period of 30 min in darkness and at 4 °C, the plates were centrifuged, washed with FACS Buffer and resuspended in 200 µl of Foxp3 Fixation/Permeabilization working solution (1:3 mix of Fixation/Permeabilization Concentrate, ThermoFisher, Cat. No.: 00-5123-43 and eBioSciences Fixation/Perm Diluent, ThermoFisher, Cat. No.: 00-5223-56). During this incubation period of 20 minutes, the Permeabilization Buffer (1:9 ThermoFisher, eBioscience™ Permeabilization Buffer 10 X, Cat. No.: 00-8333-56 + distilled water) for the next washing steps was prepared. The plates were centrifuged, the supernatant discarded and again incubated for 20 min with a 200 µl Goat Serum buffer per well (20% Goat Serum, Sigma-Aldrich, Cat. No.: G6767 + 80% Permeabilization Buffer). After another instance of centrifugation, intracellular stimulation was performed using a 100 µl/well intracellular master-mix. The intracellular master-mix contained 100 µl Permeabilization buffer, 0.2 µl antibodies (IL-2 = PE Anti-human IL-2, BioLegend, Clone MQ1-17H12, Cat. No.: 500 306, IL4 = BV 421 anti-human IL-4 BioLegend Clone MP4-25D2, Cat. No.: 500825, TNFα = Anti-human TNFα BV 605 BioLegend, Clone MAb11, BD557647, Cat. No.: 502935, type II IFNγ = BV 785 Anti-human IFNγ BioLegend Clone 4S.B3, BD/557995, Cat. No.: 502541) and 1 µl TruStain.

This step was followed up by a 30 min incubation period at 4 °C. After a washing step with the Permeabilization Buffer, the PBMCs were diluted in 200 µl of 1% PFA solution (Thermo Fisher, Cat. No.: J19943.K2) for each well. For flow-cytometry-analysis, the plates were centrifuged for 5 minutes at 400 g, room temperature, the supernatant discarded, and the cells re-suspended in 200 µl PBS in a FACS tube.

Analysis and interpretation of the ICS-panel were done after performing a background subtraction by using the results from the no peptide/Brefeldin A/DMSO stimulation as background.

An internal control was performed using a stimulation with SEB/GolgiStop/DMSO as positive control and a dual-negative stimulation (no peptide/no Brefeldin A/DMSO), which returned an overall strong reaction for SEB stimulation and a nearly zero to zero reaction in our dual-negative control.

### Immunophenotyping

The phenotype panels were performed specifically for B, natural killer (NK) and T cells using antibodies targeting surface markers by cluster of differentiation. PBMCs were used after thawing and performing a viability check using the same process described for the ICS panel. As a second step, the PBMCs were washed and stained using the same master-mix for extracellular staining as described above in the methods for the ICS. The T cell panel consisted of different antibodies targeting CD38 (= PE anti-human CD38 Antibody, BioLegend, Clone HB7, Cat. No.: 356604), CD57 (= APC anti-human CD57 Antibody, BioLegend, Clone HNK1, Cat. No.: 359610), CD45RA (= FITC anti-human CD45RA Antibody, BioLegend, Clone HI100, Cat. No.: 304106), CD185 (= PE/Cyanine7 anti-human CD185 (CXCR5) Antibody, BioLegend, Clone J252D4, Cat. No.: 356924), HLA-DR (= Brilliant Violet 421™ anti-human HLA-DR Antibody, BioLegend, Clone L243, Cat. No.: 307636), CD8 (d), CD279 (= Brilliant Violet 605™ anti-human CD279 (PD-1) Antibody, BioLegend, Clone EH12.2H7, Cat. No.: 329924), CD25 (= Brilliant Violet 650™ anti-human CD25 Antibody, BioLegend, Clone BC96, Cat. No.: 302634), CD197 (= Brilliant Violet 711™ anti-human CD197 (CCR7) Antibody, BioLegend, Clone g043H7, Cat. No.: 353228), CD127 (= Brilliant Violet 785™ anti-human CD127 (IL-7Rα) Antibody, BioLegend, Clone A019D5, Cat. No.: 351330), CD27 (= PerCP/Cyanine5.5 anti-mouse/rat/human CD27 Antibody, BioLegend, Clone LG3A10, Cat. No.: 124214), CD3, CD4 and a Life/Dead-stain. The B-Cell panel included antibodies targeting CD38, CD138 (= APC anti-human CD138 (Syndecan-1) Antibody, BioLegend, Clone DL-101, Cat. No.: 352308), CD19 (= FITC anti-human CD19 Antibody, BioLegend, Clone SJ25C1, Cat. No.: 363008), IgD (= PE/Cyanine7 anti-human IgD Antibody, BioLegend, Clone IA6-2, Cat. No.: 348210), IgG (= Brilliant Violet 421™ anti-human IgG Fc Antibody, BioLegend, Clone M1210G05, Cat. No.: 410704), CD24 (= Brilliant Violet 510™ anti-human CD24 Antibody, BioLegend, Clone ML5, Cat. No.: 311126), IgM (= Brilliant Violet 605™ anti-human IgM Antibody, BioLegend, Clone MHM-88, Cat. No.: 314524), CD20 (= Brilliant Violet 785™ anti-human CD20 Antibody, BioLegend, Clone 2H7, Cat. No.: 302355), CD27, CD3, CD21 (= PE/Dazzle™ 594 anti-human CD21 Antibody, BioLegend, Clone Bu32) and Live/Dead. For these phenotype panels an amount of at least 1 million PBMCs was used.

The NK panel was stained using CD159c (= PE anti human antibody CD159c (NKG2C), BioLegend, clone S19005E, Cat. No.: 375004), CD57, CD158 (= FITC anti-human CD158 (KIR2DL1/S1/S3/S5) Antibody, BioLegend, clone HP-MA4, Cat. No.: 339504), CD158b (= PE/Cyanine7 anti-human CD158b/j (KIR2DL2/L3/S2) Antibody, BioLegend, clone DX27, Cat. No.: 312610), CD158e (= Brilliant Violet 421™ anti-human CD158e1 (KIR3DL1, NKB1) Antibody, BioLegend, clone DX9, Cat. No.: 312714), CD56 (= Brilliant Violet 510™ anti-human CD56 (NCAM) Antibody, BioLegend, clone 5.1H11, Cat. No.: 362534), CD16 (= Brilliant Violet 605™ anti-human CD16 Antibody, BioLegend, clone 3G8, Cat. No.: 302040), CD186 (= Brilliant Violet 711™ anti-mouse CD186 (CXCR6) Antibody, BioLegend, Clone SA051D1, Cat. No.: 743601), CD90 (= Brilliant Violet 785™ anti-human CD90 (Thy1) Antibody, BioLegend, clone 5E10, Cat. No.: 328142), CD314 (= PerCP/Cyanine5.5 anti-human CD314 (NKG2D) Antibody, BioLegend, clone 1D11, Cat. No.: 320818), CD3, CD159 (= PE/Dazzle™ 594 anti-human CD159a (NKG2A) Antibody, BioLegend, clone S19004C, Cat. No.: 375122) and Live-Dead.

We performed flow cytometry analysis on the PBMCs treated in the ICS and phenotyping protocols. The samples were acquired on BD LSRFortessa Cell Analyzer and analysed with FlowJo™ v10.8 Software (BD Life Sciences).

### Data analysis

Data analysis was performed using the programming language ‘python’ and data analysis packages ‘pandas’ [44] and ‘scipy’ [45]. Visualization of the data was performed using python packages ‘matplotlib’ [46], ‘seaborn’ [47] and ‘statannotations’ [48]. Correlations between the ICS response and the serostatus as well as correlations between the serostatus and the phenotyping results were computed using the spearman coefficient [49]. Group-wise differences in the ICS response and phenotyping data were tested for significance using the Kruskal-Wallis test [50]. The results were then interpreted using Dunne’s post-hoc analysis [51]. Correction for multiple testing was performed using the Benjamini-Hochberg correction [52].

## Results

### Cohort characteristics

The full cohort consisted of a total of 174 participants from 78 households. 105 participants had a confirmed positive PCR test between March and April 2020 and/or a positive response in the serological testing against SARS-CoV-2. Household members of positive participants with a negative PCR-result and a negative antibody test were defined as exposed subjects. The control group consisted of 15 households with 33 members. All members of the control group had multiple negative SARS-CoV-2 specific PCR tests over the course of the first pandemic year and were also tested negative for SARS-CoV-2 antibodies several times. The details of the study cohort are listed in Table 1.

**Table 1:** Cohort characteristics.

Cohort	SARS-CoV-2 Positive		Exposed		Controls		All	
	All	ICS	All	ICS	All	ICS	All	ICS
**(n)**	105	50	36	16	33	10	174	76
**Age (median, range)**	38, 14-76	38, 14-70	36, 14-73	33, 14-66	41, 13-73	41, 17-63	39, 13-76	38, 14-70
**Female sex**	51 [48.6%]	27 [54%]	19 [52.8%]	8 [50%]	18 [54.5%]	7 [70%]	88 [50.6%]	42 [55.3]
**Male sex**	54 [51.4%]	23 [46%]	17 [47.2%]	8 [50%]	15 [45.5%]	3 [30%]	86 [49.4%]	34 [44.7%]
**BMI (median, range)**	21.4, 14.2-38.3	21.4, 16.4-38.3	20.9, 14.7-29.5	19.5, 14.7-26.5	21.3, 15-38.5	20.8, 15-29.4	21.3, 14.2-38.3	20.9, 14.7-38.3
**Days since infection (median, range)**	243.6, [19] 186-318	294.1, 186-301	NA	NA	NA	NA		
**Symptoms during acute infection**	81 [77.1%]	37 [74%]	2 [5.6%]	1 [6.3%]	0	0	83 [47.7%]	38 [50%]
**SEROSTATUS**								
**229E**	11 [10.5%]	6 [12%]	1 [2.8%]	0	2 [6.1%]	1 [10%]	14 [8%]	7 [9.2%]
**NL63**	39 [37.1%]	21 [42%]	13 [36.1%]	5 [31.3%]	3 [9.1%]	1 [10%]	55 [31.4%]	27 [35.5%]
**OC43**	29 [27.6%]	15 [30%]	9 [25%]	5 [31.3%]	3 [9.1%]	2 [20%]	41 [23.4%]	22 [28.9%]
**HKU1**	33 [31.4%]	17 [34%]	6 [16.7%]	4 [25%]	6 [18.2%]	3 [30%]	45 [25.7%]	24 [31.6%]
**NP-SARS-2**	74 [70.5%]	38 [76%]	0	0	0	0	74 [42.5%]	38 [50%]
**S1-SARS-2**	90 [85.7%]	41 [82%]	0	0	0	0	90 [51.7%]	41 [53.9%]
**RBD-SARS-2**	88 [83.8%]	39 [78%]	0	0	0	0	88 [50.6%]	39 [51.3%]

Demographic and serological details are shown across three groups: SARS-CoV-2 positive, exposed and controls. Additionally shown are the subgroups of participants with enough material for conduction of Intracellular Cytokine Staining (ICS) assays.

**Fig 1:**
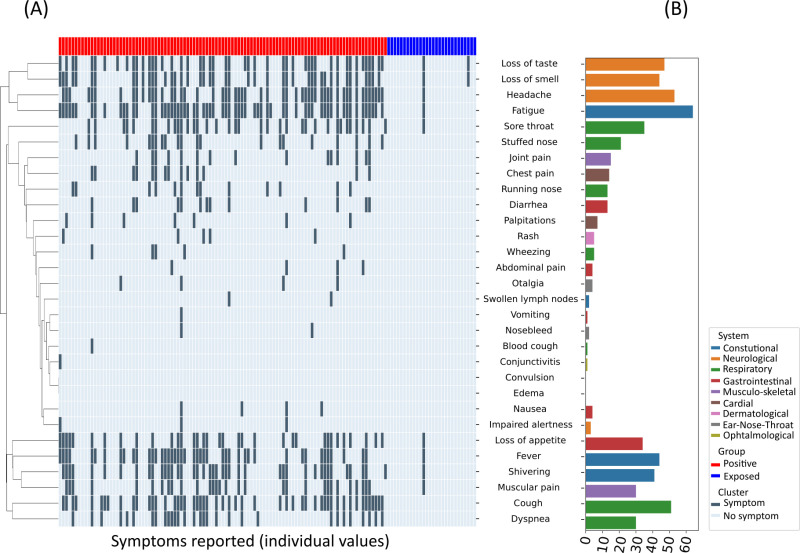
Overview of symptoms reported. (A) shows the distribution of symptoms reported by the participants in the SARS-CoV-2 positive (red, n = 105) and the exposed (blue, n = 36) group. Members of the control group did not report any symptoms. The dendrogram on the left side of the heatmap depicts two clusters of symptoms. The top cluster features symptoms commonly associated with COVID-19 disease, such as loss of smell/taste and fatigue; the bottom cluster indicates nonspecific symptoms of common respiratory infections like fever, cough, shivering or muscular pain. (B) illustrates the number of participants reporting a specific symptom. The symptoms are grouped according to the affected functional system, represented by different colours.

It depicts the three groups (SARS-CoV-2 positive, exposed and controls) of comparable age (median = 39), comparable BMI (median = 21.3) and comparable biological sex distribution. The median age and weight are lower than the German median age and mean weight [53,54]. The seropositive group is larger than both the exposed and control group. Out of all positive participants, 77.1% reported symptoms during their infection. Only 5.6% of the exposed participants reported symptoms during the infection of the index patient in their household. As there were no hospitalizations among the participants, all cases are considered mild or moderate in accordance with the ‘Coronavirus Disease 2019 (COVID-19) Treatment Guidelines NIH’ guideline [55]. Distribution and frequency of reported symptoms are shown in Fig 1, demonstrating the wide range of clinical symptoms of COVID-19. The most reported symptoms are those of a mild viral infection, including headache, fever, fatigue and sore throat. Some individuals reported dyspnea, indicating a moderate infection [55].

The ICS assay, utilizing SARS-CoV-2 specific peptides for in-vitro stimulation, was conducted on a subset of 76 participants, who provided a sufficient amount of blood samples. The ICS sub-cohort, shown in Table 1, is representative of the full cohort in terms of age, BMI, distribution of sex and reported symptoms. While all samples were collected more than 6 months post-infection, the median duration between infection and blood sample collection was greater in the ICS subset, compared to the full cohort (median = 294.1 days vs. 243.6 days). The distribution in number of group members was slightly different between the full cohort and the ICS cohort. This applied to the control group members in the full cohort (33/174 = 19%) vs the ICS cohort (10/76 = 13.2%), as well as the positive members in the full cohort (105/174 = 60.3%) vs the ICS cohort (50/76 = 65.8%).

### Serological testing reveals congruent response to SARS-CoV-2 antigens among convalescent individuals and limited cross-reactivity between different coronaviruses

**Fig 2:**
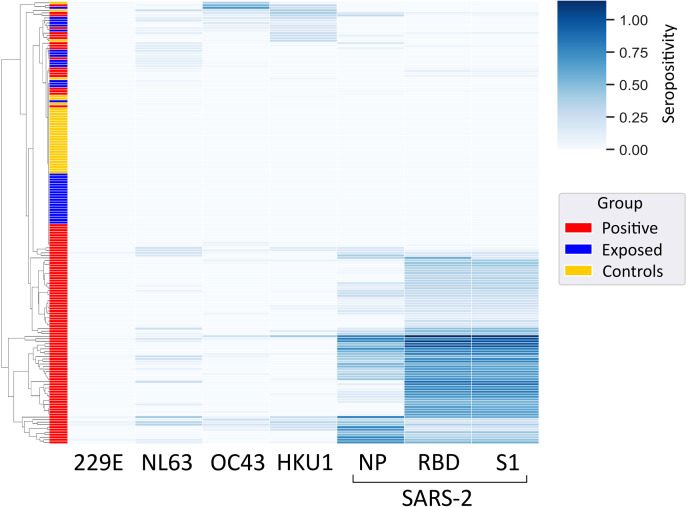
Coronavirus serostatus clustering. Shown are the results of the serological tests for SARS-CoV-2 and HCoVs clustered by the strength of response (seropositivity). The exposed (blue, n2 = 36) and control (yellow, n3 = 33) groups did – by definition – not show a serological answer against SARS-CoV-2. The positive (red, n1 = 105) group prominently features the bottom right cluster of samples that had a strong serological answer against the N, RBD and S1 SARS-CoV-2 antigens. This cluster is subdivided into smaller clusters separated by their answer to the different SARS-CoV-2-antigens.

The serostatus for HCoVs and SARS-CoV-2 in the full cohort (n = 174) is presented in Table 1 and Fig 2. The distribution of the serostatus in the full cohort is comparable to the distribution observed in the ICS sub-cohort (n = 76; S1 Fig). More than 70% of samples from recovered patients showed a clear positive result for at least one of the SARS-CoV-2 antigens.

The seroprevalence of antibodies against HCoVs was less distinct than that against SARS-CoV-2. This observation applies to all HCoVs, with prevalence rates ranging from a high of 31.4% for HCoV-NL63 to a low of 8% for HCoV-229E across all samples. The seroprevalence of HCoV antibodies also varied among the positive, exposed and control groups, with the highest rates observed in the SARS-CoV-2 positive group. Clustering the serological response – as shown in Fig 2 – revealed that the majority of samples in the convalescent group exhibited detectable responses to all tested SARS-CoV-2 antibodies (RBD-SARS-2, NP-SARS-2, S1-SARS-2). The cluster of SARS-CoV-2 positive samples could be further subdivided into three groups: one subgroup displaying responses to each of the tested SARS-CoV-2 antigens, a second subgroup showing a stronger response specifically to RBD and S1, and a third subgroup with a more pronounced response to NP compared to RBD and S1. A preservation of IgG antibodies against SARS-CoV-2 was therefore detectable in this cohort > 250 days after initial infection. Additionally, we identified a cluster of samples that responded to both beta-coronaviruses HCoV-HKU1 and HCoV-OC43. Notably, HCoV-NL63 and SARS-CoV-2 did not form a cluster with any of the other coronaviruses.

### Persistent CD4+ T cell memory subset alterations are detectable >250 days post SARS-CoV-2 infection

**Fig 3:**
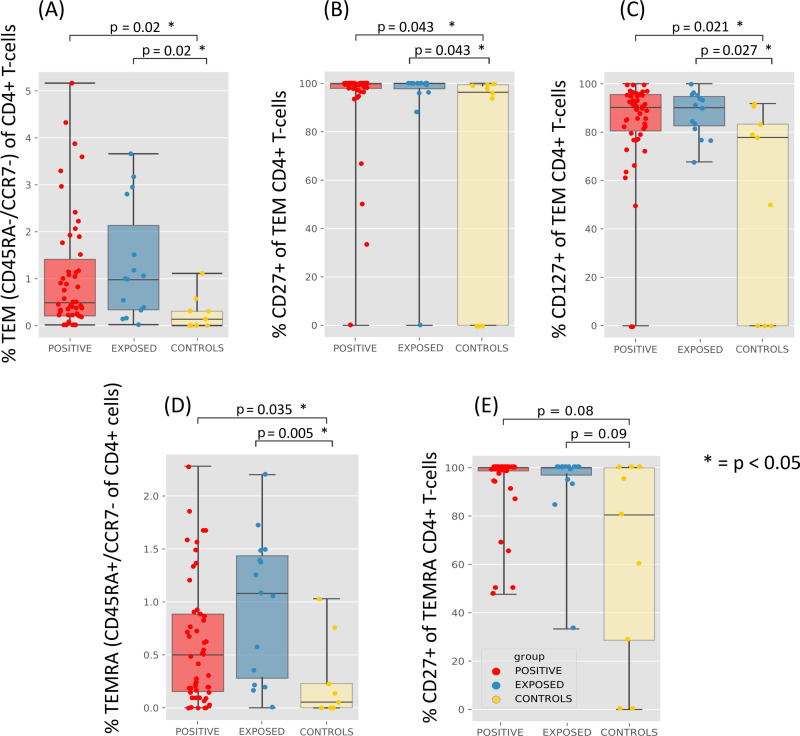
CD4+ T cell memory subset alterations after SARS-CoV-2 infection. Shown are differences in frequency of T cell subsets between the positive (red, n = 50), exposed (blue, n = 16) and control (yellow, n = 10) groups, generated by analyzing the phenotype-FACS-data. (A) to (E) depict five different subsets of effector memory CD4+ T cells: **(A)** CD4+ T_EM_ (CD45RA-/CCR7-) with its subsets CD4+ T_EM_ CD27+ (B) and CD4+ T_EM_ CD127+ (C) and **(D)** CD4+ T_EMRA_ (CD45RA+ /CCR7-) T cells with its subset CD4+ T_EMRA_ CD27+ **(E)**. Statistics were performed using the Kruskal-Wallis-test and Dunne’s post hoc test and adjusted using the Benjamini-Hochberg-correction.

Next, we performed immunophenotyping across the three groups (positive, exposed and control) to assess persisting immunological alterations more than 6 months after mild to moderate SARS-CoV-2 infection. We detected a relevant increase in the population sizes of effector memory (T_EM_) and terminally differentiated (T_EMRA_) CD4+ T cells in the positive convalescent group compared to the control group (S2 Fig). A similar increase also arose between the exposed group and the control group (Fig 3A). This difference in population size was reflected in the expression of CD4+ T_EM_ CD27+ cells (Fig 3B) and CD4+ T_EM_ CD127+ (IL-7 receptor) cells (Fig 3C) as well. Differences in the CD4+ T_EMRA_ CD27+ subset did not reach statistical significance (Fig 3E).

**Fig 4:**
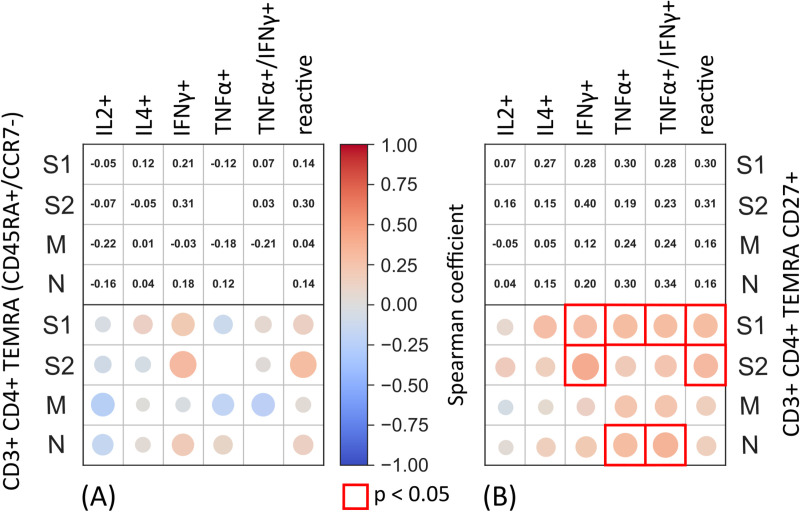
Correlation between CD4+ T_EMRA_ subsets and SARS-CoV-2-specific CD4+ T cell responses. Shown are correlations between frequency of CD4+ T_EMRA_, CD4+ T_EMRA_ CD27+ T cell subsets and SARS-CoV-2 specific CD4+ T cells responses after stimulation with one of the following peptides: S1 (Spike glycoprotein subunit 1), S2 (Spike glycoprotein subunit 2), N, M within all positive samples in the ICS sub cohort (n = 50). Antigen-specific responses are shown for: interleukin 2 and 4 (IL-2, IL-4), IFNγ, TNFα, double positive TNFα/IFNγ and the sum of TNFα and IFNγ responses (“reactive”). The upper part of both subsets shows the spearman correlation coefficient, the lower part visualizes the strength of said spearman correlation coefficient. The empty boxes for CD4+ T_EM_ cells had a correlation coefficient of 0.00 and were removed for better readability. The boxes bordered in red highlight a significant correlation coefficient (p < 0.05).

SARS-CoV-2 specific recall cytokine responses were generally low within our cohort (e.g., for IFNγ mean 0.011% of CD4 T cells (IQR 0–0.15) after background subtraction). Nevertheless, the frequencies of terminally differentiated CD4+ T cell populations (Fig 3D, 3E) positively correlated with the frequencies of SARS-CoV-2 specific CD4+ T cell responses as measured by ICS assay (Fig 4). Additionally, we found significant positive correlations between the CD4+ T_EMRA_ CD27+ population and double-positive (IFNγ+ /TNFα+) CD4+ T cell responses, when stimulated with SARS-CoV-2-S1 and N peptide-pool. In summary, we observed alterations in CD4+ T cell memory subsets that were associated with SARS-CoV-2 specific T cell responses more than 250 days post-infection.

Further, we conducted extensive immunophenotyping including T cell exhaustion and activation markers, CD8+ T cells, B cells and NK cells. We did not detect relevant differences between the three groups in the non CD4+ T cells. Alterations in mature, CD19+ /20+ memory B cell populations were observed partially in the CD27+ CD21+ and CD27- CD21+ subsets (S3 Fig). Consequently, for further analyses of adaptive immunity, we decided to focus on the antigen-specific antibodies and T cells.

## Discussion

This study presents results of a previously SARS-CoV-2 naive, pre-vaccination [56] and thus historic COVID-19 cohort. A cohort with these characteristics is not readily available at present time after administration of 980 million doses of COVID-19-vaccine in the European Union [57] and an estimated 775 million SARS-CoV-2 infections worldwide [58]. Consequently, research concerning this early stage of the COVID-19-pandemic can give insight into longevity of immune response, unbiased by later developments.

The serostatus of the participants in our cohort showed a ‘cluster of responsiveness’ between two to three SARS-CoV-2 directed antibodies (NP-SARS-2, RBD-SARS-2, S1-SARS-2), which distinguished the group of individuals with confirmed SARS-CoV-2 infection from the other groups. This is in line with previous reports of broad induction of antibodies targeting different SARS-CoV-2 antigens by natural infection [2,59]. We detected higher antibody titers against RBD- and S1 antigen than against the N (nucleocapsid) antigen, indicating a shorter persistence for the latter, as was reported previously [60]. The shorter half-life of N antibodies could explain the observed sub-clusters: Participants with a more recent infection at the time of sample collection could show a positive response against all three SARS-CoV-2 antigens. In contrast, those with a less recent infection did exhibit no or a weak reaction to the N antigen, indicating a decline in antibody levels over time [60–63]. There is also evidence that “natural” SARS-CoV-2 infections before vaccination lead to higher N antibodies, as reported by Dhakal et al. [64]. Other groups reported a half-life of N antibodies of around 85 days [65] or a correlation of antibody titre and disease severity [66]. In our cohort of “naturally” infected and mildly to moderately ill participants, we are limited in our range to estimate behaviour of antibody decline over time and thus argue for further research on this topic.

In summary, a detectable serological response against SARS-CoV-2 persists even after an extended period (median 243 days post-infection), which fits into a reported half-life of approx. 250 days [67]. Notably, our cohort consists of individuals with mild or moderate symptoms and antibody levels have been reported [66,68–70] to correlate positively with disease severity. This may further strengthen the observation of a persisting antibody response, as it is detectable even in a cohort of mildly/moderately ill individuals. It is unlikely that re-infections occurred between the initial positive test for SARS-CoV-2 and blood sampling, given the low risk of re-infection within the first six months post-infection [68,69]. However, this cannot be formally excluded. In any case, we do not regard the detected responses as robust enough to suggest a secondary, more recent infection with SARS-CoV-2 within the cohort studied.

The serostatus for the seasonal 'common cold coronaviruses (CCCs) or HCoVs (229E, HKU1, NL63, OC43) indicates a lower seroprevalence than previously reported in other cohorts from Japan [70], Canada [71] and the USA [72]. While the cited data vary by region and are not easily comparable to German demographics, the overall prevalence rates of the four HCoVs (approx. 70% to 90%), are considerably higher than the prevalence rates observed in our study (8% 229E to 31.4% NL63). Studies from Germany report a PCR-based prevalence of HCoVs of less than 10% in late 2019 and early 2020. They also show a notable decline after the onset of non-pharmaceutical interventions (NPIs), though the infrequency of such analyses in Germany renders a proper benchmark difficult [73,74].

The low seroprevalence observed here may be attributed to a combination of two factors: First, the antibody response against the HCoVs is reported to be volatile and relatively short-lasting [75,76]. Second, a reduction in infections with other seasonal respiratory viruses occured, including influenza and HCoVs, attributable to NPI [77,78] which were implemented in Bavaria as early as March 2020 [79].

A previously reported weak cross-reactivity between antibodies targeting SARS-CoV-2 and HCoV-OC43 [80] could not be replicated in this cohort. Similarly, we did not detect cross-reactivity between antibodies against any of the CCCs and SARS-CoV-2. This may be attributed to the low prevalence of antibodies against CCCs in this cohort. It may also add up to the conflicting results regarding evidence of cross-reactive antibodies [81]. While Sealy et al. [82] reported mixed results on cross-reactivity, Murray et al. [83] found cross-reactivity not influencing the clinical outcome of a SARS-CoV-2 infection. Other groups like Ortega et al. [84] or Sagar et al. [85] reported a protective effect of pre-existing HCoV antibodies on an infection with SARS-CoV-2. Population groups with a reportedly higher level of cross-reactivity, such as children and adolescents were not included in this study [86,87].

CD4+ T cells can be divided into naïve (CD4+ CD45RA+ CCR7+), effector memory (CD4+ CD45RA- CCR7-), central memory (CD4+ CD45RA- CCR7+) and T_EMRA_ (CD4+ CD45RA+ CCR-) cells [16,67]. Effector memory populations, including CD4+ T_EM_ and CD4+ T_EMRA_ cells expressing the IL-7 receptor CD127 [88], are important components of the adaptive immune system in mediating recall antiviral immunity [89–92]. We observed alterations in CD4+ cell memory populations in our cohort correlating with SARS-CoV-2 specific cytokine responses of IFNγ and TNFα. IFNγ is an effector cytokine secreted by T cells and NK cells mediating antiviral function, including in respiratory infections like SARS-CoV-2 [1,16]. These cytokines are known to be key effectors in the context of SARS-CoV-2 immune response [93–95]. Even though this correlation provides an indirect detection of SARS-CoV-2-specific T cells they suggest a detectable SARS-CoV-2 specific CD4+ T cell response up to 300 days after an acute infection (median of the ICS cohort: 294.1 days). However, due to the indirect detection our results may approximate the real effect. These results fit into the reported timeframe of 6–10 months of robust to detectable T cell responses after SARS-CoV-2 infection as outlined by various studies [14,35,62,96]. The changes observed in the distribution of CD4+ T cell populations also apply to the exposed group, suggesting that household exposure to the virus can induce an immune response even in healthy individuals, as other studies have suggested [97,98]. In view of the development of vaccines targeting the S antigens [99], our results – showing a significant correlation of increased IFNγ and TNFα secretion and increased T cell response, when stimulated with SARS-CoV-2-S1 (Fig 4) – are fitting as a potential mechanism for induction of immunity through vaccination.

In contrast to other studies reporting prolonged immune responses for CD8+ T_EMRA_ cells [67,100–102], B cells Dan et al. [62] or NK cells [103], SARS-CoV-2-specific immunity in our cohort was limited to CD4 memory populations. This might be due to our cohort only containing participants with mild to moderate disease, as others have found expanded NK cell subsets in severely ill patients [104]. Therefore, we think further research is needed to further distinguish NK cell answers against SARS-CoV-2 while correcting for disease severity. Regarding potential differences in B cell populations (S3 Fig) and their role in antibody secretion after infection or vaccination [99] further research is needed.

Limitations of this study are the imbalance in the number of samples between the positive versus the exposed and controls group as well as the smaller sample size in the ICS panel compared to the full cohort. This was due to sample availability and may have compromised the statistical comparison between the groups. This could lead to over- or underestimations of the real values. Nevertheless, we observed similar results in the overall cohort and the smaller ICS subset, supporting the reliability of our findings and interpretations.

## Supporting information

S1 Fig Coronavirus serostatus clustering ICS cohort. Shown are the results of the serological tests for SARS-CoV2 and HCoVs clustered by the strength of response (seropositivity) in the ICS subset. The exposed (blue) and control (yellow) groups did – by definition – not show a signally serological answer against SARS-CoV-2. The positive (red) group prominently features the bottom right cluster of samples that had a strong serological answer against SARS-CoV-2. This cluster is subdivided into smaller clusters separated by their answer to the different SARS-CoV-2-antigens.
(TIF)

S2 Fig Example of FACS-gating strategy. Example of the FACS-gating strategy used to determine the extents of the T cell populations. Shown is the gating procedure, used to determine subpopulations of the PBMC population (lymphocytes - > single cells - > alive cells - > CD3+ cells) (A). Subfigure (B) shows the differentiation for CD4+ T cells. Subfigure (C) shows the differentiation between CCR7 and CD45RA CD4+ T cell populations used in the phenotyping protocol. Subfigures (D) show the IFNγ and TNFα producing CD4+ T cell subsets. (D) depicts results of stimulation with SARS-CoV-2 peptides M, N, S1 and S2 as well as the stimulation controls (SEB and control).
(TIF)

S3 Fig Selected B cell memory subset alterations after SARS-CoV-2 infection. Shown are CD27+ CD21+ (A) and CD27- CD21+ (B) subsets of mature (CD19+ /CD20+) B cells in comparison between the positive (red), exposed (blue) and control group (yellow). Statistics were performed using the Kruskal-Wallis-test and Dunne’s post hoc test and adjusted using the Benjamini-Hochberg-correction.
(TIF)

S1 File Data Sheet: ICS-, Phenotyping, Symptom- and Serodata. (XLSX)

## References

[1] Huang C, Wang Y, Li X, Ren L, Zhao J, Hu Y, et al. Clinical features of patients infected with 2019 novel coronavirus in Wuhan, China. Lancet. 2020;395(10223):497–506. doi: 10.1016/S0140-6736(20)30183-5 31986264 PMC7159299

[2] Lapuente D, Winkler TH, Tenbusch M. B-cell and antibody responses to SARS-CoV-2: infection, vaccination, and hybrid immunity. Cell Mol Immunol. 2024;21(2):144–58. doi: 10.1038/s41423-023-01095-w 37945737 PMC10805925

[3] Moss P. The T cell immune response against SARS-CoV-2. Nat Immunol. 2022;23(2):186–93. doi: 10.1038/s41590-021-01122-w 35105982

[4] Nesteruk I. Endemic characteristics of SARS-CoV-2 infection. Sci Rep. 2023;13(1):14841. doi: 10.1038/s41598-023-41841-8 37684338 PMC10491781

[5] WHO - World Health Organisation. Coronavirus disease (COVID-19) pandemic 2024. Available from: https://www.who.int/europe/emergencies/situations/covid-19

[6] Su S, Wong G, Shi W, Liu J, Lai ACK, Zhou J, et al. Epidemiology, Genetic Recombination, and Pathogenesis of Coronaviruses. Trends Microbiol. 2016;24(6):490–502. 27012512 10.1016/j.tim.2016.03.003 PMC7125511

[7] Chen N, Zhou M, Dong X, Qu J, Gong F, Han Y, et al. Epidemiological and clinical characteristics of 99 cases of 2019 novel coronavirus pneumonia in Wuhan, China: a descriptive study. Lancet. 2020;395(10223):507–13. doi: 10.1016/S0140-6736(20)30211-7 32007143 PMC7135076

[8] Ruan Q, Yang K, Wang W, Jiang L, Song J. Clinical predictors of mortality due to COVID-19 based on an analysis of data of 150 patients from Wuhan, China. Intensive Care Med. 2020;46(5):846–8. doi: 10.1007/s00134-020-05991-x 32125452 PMC7080116

[9] Bunders MJ, Altfeld M. Implications of Sex Differences in Immunity for SARS-CoV-2 Pathogenesis and Design of Therapeutic Interventions. Immunity. 2020;53(3):487–95. doi: 10.1016/j.immuni.2020.08.003 32853545 PMC7430299

[10] Yehia BR, Winegar A, Fogel R, Fakih M, Ottenbacher A, Jesser C, et al. Association of Race With Mortality Among Patients Hospitalized With Coronavirus Disease 2019 (COVID-19) at 92 US Hospitals. JAMA Netw Open. 2020;3(8):e2018039. doi: 10.1001/jamanetworkopen.2020.18039 32809033 PMC7435340

[11] Di Fusco M, Lin J, Vaghela S, Lingohr-Smith M, Nguyen JL, Scassellati Sforzolini T, et al. COVID-19 vaccine effectiveness among immunocompromised populations: a targeted literature review of real-world studies. Expert Rev Vaccines. 2022;21(4):435–51. doi: 10.1080/14760584.2022.2035222 35112973 PMC8862165

[12] Chi WY, Li YD, Huang HC, Chan TEH, Chow SY, Su JH, et al. COVID-19 vaccine update: vaccine effectiveness, SARS-CoV-2 variants, boosters, adverse effects, and immune correlates of protection. Journal of Biomedical Science. 2022;29(1). 10.1186/s12929-022-00853-8 PMC9569411 36243868

[13] Peng Y, Mentzer AJ, Liu G, Yao X, Yin Z, Dong D, et al. Broad and strong memory CD4+ and CD8+ T cells induced by SARS-CoV-2 in UK convalescent individuals following COVID-19. Nat Immunol. 2020;21(11):1336–45. doi: 10.1038/s41590-020-0782-6 32887977 PMC7611020

[14] Jung JH, Rha M-S, Sa M, Choi HK, Jeon JH, Seok H, et al. SARS-CoV-2-specific T cell memory is sustained in COVID-19 convalescent patients for 10 months with successful development of stem cell-like memory T cells. Nat Commun. 2021;12(1):4043. doi: 10.1038/s41467-021-24377-1 34193870 PMC8245549

[15] Loyal L, Braun J, Henze L, Kruse B, Dingeldey M, Reimer U, et al. Cross-reactive CD4+ T cells enhance SARS-CoV-2 immune responses upon infection and vaccination. Science. 2021;374(6564):eabh1823. doi: 10.1126/science.abh1823 34465633 PMC10026850

[16] Brown B, Ojha V, Fricke I, Al-Sheboul SA, Imarogbe C, Gravier T, et al. Innate and Adaptive Immunity during SARS-CoV-2 Infection: Biomolecular Cellular Markers and Mechanisms. Vaccines (Basel). 2023;11(2):408. doi: 10.3390/vaccines11020408 36851285 PMC9962967

[17] Eggenhuizen PJ, Ooi JD. The Influence of Cross-Reactive T Cells in COVID-19. Biomedicines. 2024;12(3):564. doi: 10.3390/biomedicines12030564 38540178 PMC10967880

[18] Abela IA, Schwarzmüller M, Ulyte A, Radtke T, Haile SR, Ammann P, et al. Cross-protective HCoV immunity reduces symptom development during SARS-CoV-2 infection. mBio. 2024;15(2):e0272223. doi: 10.1128/mbio.02722-23 38270455 PMC10865973

[19] Liu DX, Liang JQ, Fung TS. Human Coronavirus-229E, -OC43, -NL63, and -HKU1 (Coronaviridae). Encyclopedia of Virology. 2021:428–40.

[20] Mateus J, Grifoni A, Tarke A, Sidney J, Ramirez SI, Dan JM, et al. Selective and cross-reactive SARS-CoV-2 T cell epitopes in unexposed humans. Science. 2020;370(6512):89–94. doi: 10.1126/science.abd3871 32753554 PMC7574914

[21] Braun J, Loyal L, Frentsch M, Wendisch D, Georg P, Kurth F, et al. SARS-CoV-2-reactive T cells in healthy donors and patients with COVID-19. Nature. 2020;587(7833):270–4. doi: 10.1038/s41586-020-2598-9 32726801

[22] Grobben M, Straten DVK, Brouwer JP, Brinkkemper M, Maisonnasse P, Dereuddre-Bosquet N, et al. Cross-reactive antibodies after SARS-CoV-2 infection and vaccination. eLife. 2021;10. 10.7554/eLife.70330 PMC8610423 34812143

[23] Tarke A, Sidney J, Kidd CK, Dan JM, Ramirez SI, Yu ED, et al. Comprehensive analysis of T cell immunodominance and immunoprevalence of SARS-CoV-2 epitopes in COVID-19 cases. Cell Reports Medicine. 2021;2(2):100204. 33521695 10.1016/j.xcrm.2021.100204 PMC7837622

[24] Grifoni A, Weiskopf D, Ramirez SI, Mateus J, Dan JM, Moderbacher CR, et al. Targets of T Cell Responses to SARS-CoV-2 Coronavirus in Humans with COVID-19 Disease and Unexposed Individuals. Cell. 2020;181(7):1489-1501.e15. doi: 10.1016/j.cell.2020.05.015 32473127 PMC7237901

[25] Grifoni A, Sidney J, Vita R, Peters B, Crotty S, Weiskopf D, et al. SARS-CoV-2 human T cell epitopes: Adaptive immune response against COVID-19. Cell Host Microbe. 2021;29(7):1076–92. 34237248 10.1016/j.chom.2021.05.010 PMC8139264

[26] Chandrashekar A, Liu J, Martinot AJ, McMahan K, Mercado NB, Peter L, et al. SARS-CoV-2 infection protects against rechallenge in rhesus macaques. Science. 2020;369(6505):812–7. 32434946 10.1126/science.abc4776 PMC7243369

[27] Gilbert PB, Donis RO, Koup RA, Fong Y, Plotkin SA, Follmann D. A Covid-19 Milestone Attained - A Correlate of Protection for Vaccines. N Engl J Med. 2022;387(24):2203–6. doi: 10.1056/NEJMp2211314 36507702

[28] Lasrado N, Collier A-RY, Miller J, Hachmann NP, Liu J, Anand T, et al. Waning immunity and IgG4 responses following bivalent mRNA boosting. Sci Adv. 2024;10(8):eadj9945. doi: 10.1126/sciadv.adj9945 38394195 PMC10889350

[29] Nesamari R, Omondi MA, Baguma R, Höft MA, Ngomti A, Nkayi AA, et al. Post-pandemic memory T cell response to SARS-CoV-2 is durable, broadly targeted, and cross-reactive to the hypermutated BA.2.86 variant. Cell Host Microbe. 2024;32(2):162–9.e3. 38211583 10.1016/j.chom.2023.12.003 PMC10901529

[30] Luckheeram RV, Zhou R, Verma AD, Xia B. CD4⁺T cells: differentiation and functions. Clin Dev Immunol. 2012;2012:925135. doi: 10.1155/2012/925135 22474485 PMC3312336

[31] Zhang N, Bevan MJ. CD8(+) T cells: foot soldiers of the immune system. Immunity. 2011;35(2):161–8. doi: 10.1016/j.immuni.2011.07.010 21867926 PMC3303224

[32] Schilling J, Buda S, Fischer M, Goerlitz L, Grote U, Haas W, et al. Retrospektive Phaseneinteilung der COVID-19-Pandemie in Deutschland bis Februar 2021. 2021;15:3–12.

[33] Pouwels KB, House T, Pritchard E, Robotham JV, Birrell PJ, Gelman A, et al. Community prevalence of SARS-CoV-2 in England from April to November, 2020: results from the ONS Coronavirus Infection Survey. Lancet Public Health. 2021;6(1):e30–8. doi: 10.1016/S2468-2667(20)30282-6 33308423 PMC7786000

[34] Hodcroft EB, Zuber M, Nadeau S, Vaughan TG, Crawford KHD, Althaus CL, et al. Spread of a SARS-CoV-2 variant through Europe in the summer of 2020. Nature. 2021;595(7869):707–12. doi: 10.1038/s41586-021-03677-y 34098568

[35] Brand I, Gilberg L, Bruger J, Garí M, Wieser A, Eser TM, et al. Broad T Cell Targeting of Structural Proteins After SARS-CoV-2 Infection: High Throughput Assessment of T Cell Reactivity Using an Automated Interferon Gamma Release Assay. Frontiers in Immunology. 2021;12(1825). 10.3389/fimmu.2021.688436 PMC8173205 34093595

[36] Hartung C, Lerer A, Anokwa Y, Tseng C, Brunette W, Borriello G. Open data kit: tools to build information services for developing regions. In: Proceedings of the 4th ACM/IEEE International Conference on Information and Communication Technologies and Development. London, United Kingdom: Association for Computing Machinery; 2010. p. Article 18.

[37] Pritsch M, Radon K, Bakuli A, Le Gleut R, Olbrich L, Guggenbüehl Noller JM, et al. Prevalence and Risk Factors of Infection in the Representative COVID-19 Cohort Munich. Int J Environ Res Public Health. 2021;18(7):3572. doi: 10.3390/ijerph18073572 33808249 PMC8038115

[38] Radon K, Bakuli A, Pütz P, Le Gleut R, Guggenbuehl Noller JM, Olbrich L, et al. From first to second wave: follow-up of the prospective COVID-19 cohort (KoCo19) in Munich (Germany). BMC Infect Dis. 2021;21(1):925. doi: 10.1186/s12879-021-06589-4 34493217 PMC8423599

[39] Dugas M, Grote-Westrick T, Vollenberg R, Lorentzen E, Brix T, Schmidt H, et al. Less severe course of COVID-19 is associated with elevated levels of antibodies against seasonal human coronaviruses OC43 and HKU1 (HCoV OC43, HCoV HKU1). Int J Infect Dis. 2021;105:304–6. doi: 10.1016/j.ijid.2021.02.085 33636357 PMC7901274

[40] Gupta R, Gupta P, Wang S, Melnykov A, Jiang Q, Seth A, et al. Ultrasensitive lateral-flow assays via plasmonically active antibody-conjugated fluorescent nanoparticles. Nat Biomed Eng. 2023;7(12):1556–70. doi: 10.1038/s41551-022-01001-1 36732621 PMC12723709

[41] Baker AN, Muguruza AR, Richards S-J, Georgiou PG, Goetz S, Walker M, et al. Lateral Flow Glyco-Assays for the Rapid and Low-Cost Detection of Lectins-Polymeric Linkers and Particle Engineering Are Essential for Selectivity and Performance. Adv Healthc Mater. 2022;11(4):e2101784. doi: 10.1002/adhm.202101784 34747143 PMC7612396

[42] Schneider CA, Rasband WS, Eliceiri KW. NIH Image to ImageJ: 25 years of image analysis. Nat Methods. 2012;9(7):671–5. doi: 10.1038/nmeth.2089 22930834 PMC5554542

[43] Altschul SF, Gish W, Miller W, Myers EW, Lipman DJ. Basic local alignment search tool. J Mol Biol. 1990;215(3):403–10. doi: 10.1016/S0022-2836(05)80360-2 2231712

[44] Tpdtp-dpPZ. 2024. pandas-dev/pandas: Pandas.pandas-dev.

[45] Virtanen P, Gommers R, Oliphant TE, Haberland M, Reddy T, Cournapeau D, et al. SciPy 1.0: fundamental algorithms for scientific computing in Python. Nat Methods. 2020;17(3):261–72. doi: 10.1038/s41592-019-0686-2 32015543 PMC7056644

[46] Hunter JD. Matplotlib: A 2D Graphics Environment. Comput Sci Eng. 2007;9(3):90–5. doi: 10.1109/mcse.2007.55

[47] Waskom M. seaborn: statistical data visualization. J Open Sour Softw. 2021;6(60):3021. doi: 10.21105/joss.03021

[48] Florian C, Marc W, Dariusz I, Emerson H, Marcin M, Joseph L, et al. u. a. trevismd/statannotations: v0.5. Zenodo. 2022.

[49] Spearman C. The Proof and Measurement of Association between Two Things. American Journal of Psychology. 1904;15(1):72. doi: 10.2307/1412159 3322052

[50] Kruskal WH, Wallis WA. Use of Ranks in One-Criterion Variance Analysis. Journal of the American Statistical Association. 1952;47(260):583–621. doi: 10.1080/01621459.1952.10483441

[51] Dunn OJ. Multiple Comparisons Using Rank Sums. Technometrics. 1964;6(3):241–52. doi: 10.1080/00401706.1964.10490181

[52] Benjamini Y, Hochberg Y. Controlling the False Discovery Rate: A Practical and Powerful Approach to Multiple Testing. Journal of the Royal Statistical Society Series B: Statistical Methodology. 1995;57(1):289–300. doi: 10.1111/j.2517-6161.1995.tb02031.x

[53] DeSTATIS - statistisches Bundesamt Deutschland. Körpermaße der Bevölkerung nach Altersgruppen 2021 (Endergebnisse) Stand 27. März 2023. Available from: https://www.destatis.de/DE/Themen/Gesellschaft-Umwelt/Gesundheit/Gesundheitszustand-Relevantes-Verhalten/Tabellen/koerpermasse-insgesamt.html

[54] BiB - Bundesinstitut für Bevölkerungsforschung. Medianalter der Bevölkerung (1950-2070). Available from: https://www.bib.bund.de/Permalink.html?cms_permaid=1217858

[55] NIH - National Institutes of Health. Clinical Spectrum of SARS-CoV-2 Infection 2024. Available from: https://web.archive.org/web/20240807005826/https://www.covid19treatmentguidelines.nih.gov/overview/clinical-spectrum/

[56] Impfboard Deutschland. Übersicht zum Impfstatus 2023. Available from: https://impfdashboard.de/

[57] European Centre for Disease Prevention and Control (ECDC). COVID-19 Vaccine Tracker 2023. Available from: https://vaccinetracker.ecdc.europa.eu/public/extensions/COVID-19/vaccine-tracker.html#uptake-tab

[58] WHO - World Health Organisation. COVID-19 Cases, World 2024. Available from: https://data.who.int/dashboards/covid19/cases

[59] Ju B, Zhang Q, Wang Z, Aw ZQ, Chen P, Zhou B, et al. Infection with wild-type SARS-CoV-2 elicits broadly neutralizing and protective antibodies against omicron subvariants. Nat Immunol. 2023;24(4):690–9. doi: 10.1038/s41590-023-01449-6 36914890 PMC10063446

[60] Grandjean L, Saso A, Torres Ortiz A, Lam T, Hatcher J, Thistlethwayte R, et al. Long-Term Persistence of Spike Protein Antibody and Predictive Modeling of Antibody Dynamics After Infection With Severe Acute Respiratory Syndrome Coronavirus 2. Clin Infect Dis. 2022;74(7):1220–9. doi: 10.1093/cid/ciab607 34218284 PMC8994590

[61] Yamayoshi S, Yasuhara A, Ito M, Akasaka O, Nakamura M, Nakachi I, et al. Antibody titers against SARS-CoV-2 decline, but do not disappear for several months. EClinicalMedicine. 2021;32:100734. doi: 10.1016/j.eclinm.2021.100734 33589882 PMC7877219

[62] Dan JM, Mateus J, Kato Y, Hastie KM, Yu ED, Faliti CE, et al. Immunological memory to SARS-CoV-2 assessed for up to 8 months after infection. Science. 2021;371(6529):eabf4063. doi: 10.1126/science.abf4063 33408181 PMC7919858

[63] Lumley SF, Wei J, O’Donnell D, Stoesser NE, Matthews PC, Howarth A, et al. The Duration, Dynamics, and Determinants of Severe Acute Respiratory Syndrome Coronavirus 2 (SARS-CoV-2) Antibody Responses in Individual Healthcare Workers. Clin Infect Dis. 2021;73(3):e699–709. doi: 10.1093/cid/ciab004 33400782 PMC7929225

[64] Dhakal S, Yu T, Yin A, Pisanic N, Demko ZO, Antar AAR, et al. Reconsideration of Antinucleocapsid IgG Antibody as a Marker of SARS-CoV-2 Infection Postvaccination for Mild COVID-19 Patients. Open Forum Infectious Diseases. 2023;10(1). 10.1093/ofid/ofac677 PMC9835753 36655185

[65] Van Elslande J, Gruwier L, Godderis L, Vermeersch P. Estimated Half-Life of SARS-CoV-2 Anti-Spike Antibodies More Than Double the Half-Life of Anti-nucleocapsid Antibodies in Healthcare Workers. Clin Infect Dis. 2021;73(12):2366–8. doi: 10.1093/cid/ciab219 33693643 PMC7989510

[66] Chansaenroj J, Yorsaeng R, Posuwan N, Puenpa J, Wanlapakorn N, Sudhinaraset N, et al. Long-term specific IgG response to SARS-CoV-2 nucleocapsid protein in recovered COVID-19 patients. Sci Rep. 2021;11(1):23216. doi: 10.1038/s41598-021-02659-4 34853374 PMC8636620

[67] Cohen KW, Linderman SL, Moodie Z, Czartoski J, Lai L, Mantus G, et al. Longitudinal analysis shows durable and broad immune memory after SARS-CoV-2 infection with persisting antibody responses and memory B and T cells. Cell Reports Medicine. 2021;2(7). 10.1016/j.xcrm.2021.100354 PMC8253687 34250512

[68] Stein C, Nassereldine H, Sorensen RJD, Amlag JO, Bisignano C, Byrne S, et al.; COVID-19 Forecasting Team. Past SARS-CoV-2 infection protection against re-infection: a systematic review and meta-analysis. Lancet. 2023;401(10379):833–42. doi: 10.1016/S0140-6736(22)02465-5 36930674 PMC9998097

[69] Wang Z, Muecksch F, Schaefer-Babajew D, Finkin S, Viant C, Gaebler C, et al. Naturally enhanced neutralizing breadth against SARS-CoV-2 one year after infection. Nature. 2021;595(7867):426–31. doi: 10.1038/s41586-021-03696-9 34126625 PMC8277577

[70] Imai K, Matsuoka M, Tabata S, Kitagawa Y, Nagura-Ikeda M, Kubota K, et al. Cross-reactive humoral immune responses against seasonal human coronaviruses in COVID-19 patients with different disease severities. Int J Infect Dis. 2021;111:68–75. doi: 10.1016/j.ijid.2021.08.026 34407480 PMC8364517

[71] Galipeau Y, Siragam V, Laroche G, Marion E, Greig M, McGuinty M, et al. Relative Ratios of Human Seasonal Coronavirus Antibodies Predict the Efficiency of Cross-Neutralization of SARS-CoV-2 Spike Binding to ACE2. EBioMedicine. 2021;74:103700. doi: 10.1016/j.ebiom.2021.103700 34861490 PMC8629681

[72] Gorse GJ, Patel GB, Vitale JN, O’Connor TZ. Prevalence of antibodies to four human coronaviruses is lower in nasal secretions than in serum. Clinical and Vaccine Immunology. 2010;17(12):1875–80. 20943876 10.1128/CVI.00278-10 PMC3008199

[73] Robert-Koch-Institut (RKI). Epidemiologisches Bulletin Ausgabe 22/2022 2022. Available from: https://www.rki.de/DE/Aktuelles/Publikationen/Epidemiologisches-Bulletin/2022/22_22.pdf?__blob=publicationFile&v=1

[74] Biere B, Oh DY, Wolff T, Dürrwald R. Surveillance of endemic human coronaviruses in Germany, 2019/2020. The Lancet Regional Health - Europe. 2021;11:100262. 34751265 10.1016/j.lanepe.2021.100262 PMC8566015

[75] Edridge AWD, Kaczorowska J, Hoste ACR, Bakker M, Klein M, Loens K, et al. Seasonal coronavirus protective immunity is short-lasting. Nat Med. 2020;26(11):1691–3. doi: 10.1038/s41591-020-1083-1 32929268

[76] Callow KA, Parry HF, Sergeant M, Tyrrell DAJ. The time course of the immune response to experimental coronavirus infection of man. Epidemiology and Infection. 1990;105(2):435–46. 2170159 10.1017/s0950268800048019 PMC2271881

[77] Jones N. How COVID-19 is changing the cold and flu season. Nature. 2020;588(7838):388–90. doi: 10.1038/d41586-020-03519-3 33324005

[78] Chow EJ, Uyeki TM, Chu HY. The effects of the COVID-19 pandemic on community respiratory virus activity. Nature Reviews Microbiology. 2022. 10.1038/s41579-022-00807-9 PMC9574826 36253478

[79] Bayerisches Landesamt für Statistik. Einflüsse der SARS-CoV 2-Pandemie seit 2020 auf die Beherbergungsstatistik in Bayern. Available from: https://www.statistik.bayern.de/mam/statistik/wirtschaft_handel/tourismus/g41003_202105_zeitstrahl_sars_cov2_pandemie_07_05_2021.pdf

[80] Hicks J, Klumpp-Thomas C, Kalish H, Shunmugavel A, Mehalko J, Denson J-P, et al. Serologic Cross-Reactivity of SARS-CoV-2 with Endemic and Seasonal Betacoronaviruses. J Clin Immunol. 2021;41(5):906–13. doi: 10.1007/s10875-021-00997-6 33725211 PMC7962425

[81] Merad M, Blish CA, Sallusto F, Iwasaki A. The immunology and immunopathology of COVID-19. Science. 2022;375(6585):1122–7. doi: 10.1126/science.abm8108 35271343 PMC12828912

[82] Sealy RE, Hurwitz JL. Cross-Reactive Immune Responses toward the Common Cold Human Coronaviruses and Severe Acute Respiratory Syndrome Coronavirus 2 (SARS-CoV-2): Mini-Review and a Murine Study. Microorganisms. 2021;9(8):1643. doi: 10.3390/microorganisms9081643 34442723 PMC8398386

[83] Murray SM, Ansari AM, Frater J, Klenerman P, Dunachie S, Barnes E, et al. The impact of pre-existing cross-reactive immunity on SARS-CoV-2 infection and vaccine responses. Nat Rev Immunol. 2023;23(5):304–16. doi: 10.1038/s41577-022-00809-x 36539527 PMC9765363

[84] Ortega N, Ribes M, Vidal M, Rubio R, Aguilar R, Williams S, et al. Seven-month kinetics of SARS-CoV-2 antibodies and role of pre-existing antibodies to human coronaviruses. Nat Commun. 2021;12(1):4740. doi: 10.1038/s41467-021-24979-9 34362897 PMC8346582

[85] Sagar M, Reifler K, Rossi M, Miller NS, Sinha P, White LF, et al. Recent endemic coronavirus infection is associated with less-severe COVID-19. J Clin Invest. 2021;131(1):e143380. doi: 10.1172/JCI143380 32997649 PMC7773342

[86] Ng KW, Faulkner N, Cornish GH, Rosa A, Harvey R, Hussain S, et al. Preexisting and de novo humoral immunity to SARS-CoV-2 in humans. Science. 2020;370(6522):1339–43. doi: 10.1126/science.abe1107 33159009 PMC7857411

[87] Anderson EM, Goodwin EC, Verma A, Arevalo CP, Bolton MJ, Weirick ME, et al. Seasonal human coronavirus antibodies are boosted upon SARS-CoV-2 infection but not associated with protection. Cell. 2021;184(7):1858-1864.e10. doi: 10.1016/j.cell.2021.02.010 33631096 PMC7871851

[88] Gattinoni L, Lugli E, Ji Y, Pos Z, Paulos CM, Quigley MF, et al. A human memory T cell subset with stem cell-like properties. Nat Med. 2011;17(10):1290–7. doi: 10.1038/nm.2446 21926977 PMC3192229

[89] Lilleri D, Fornara C, Revello MG, Gerna G. Human cytomegalovirus-specific memory CD8+ and CD4+ T cell differentiation after primary infection. J Infect Dis. 2008;198(4):536–43. doi: 10.1086/590118 18590456

[90] Tian Y, Babor M, Lane J, Schulten V, Patil VS, Seumois G, et al. Unique phenotypes and clonal expansions of human CD4 effector memory T cells re-expressing CD45RA. Nat Commun. 2017;8(1):1473. doi: 10.1038/s41467-017-01728-5 29133794 PMC5684192

[91] Benito JM, Zabay JM, Gil J, Bermejo M, Escudero A, Sánchez E, et al. Quantitative alterations of the functionally distinct subsets of CD4 and CD8 T lymphocytes in asymptomatic HIV infection: changes in the expression of CD45RO, CD45RA, CD11b, CD38, HLA-DR, and CD25 antigens. J Acquir Immune Defic Syndr Hum Retrovirol. 1997;14(2):128–35. doi: 10.1097/00042560-199702010-00005 9052721

[92] Bofill M, Mocroft A, Lipman M, Medina E, Borthwick NJ, Sabin CA, et al. Increased numbers of primed activated CD8+CD38+CD45RO+ T cells predict the decline of CD4+ T cells in HIV-1-infected patients. AIDS. 1996;10(8):827–34. doi: 10.1097/00002030-199607000-00005 8828739

[93] Tan AT, Linster M, Tan CW, Le Bert N, Chia WN, Kunasegaran K, et al. Early induction of functional SARS-CoV-2-specific T cells associates with rapid viral clearance and mild disease in COVID-19 patients. Cell Rep. 2021;34(6):108728. doi: 10.1016/j.celrep.2021.108728 33516277 PMC7826084

[94] Karki R, Sharma BR, Tuladhar S, Williams EP, Zalduondo L, Samir P, et al. Synergism of TNF-α and IFN-γ Triggers Inflammatory Cell Death, Tissue Damage, and Mortality in SARS-CoV-2 Infection and Cytokine Shock Syndromes. Cell. 2021;184(1):149-168.e17. doi: 10.1016/j.cell.2020.11.025 33278357 PMC7674074

[95] Banerjee A, El-Sayes N, Budylowski P, Jacob RA, Richard D, Maan H, et al. Experimental and natural evidence of SARS-CoV-2-infection-induced activation of type I interferon responses. iScience. 2021;24(5):102477. doi: 10.1016/j.isci.2021.102477 33937724 PMC8074517

[96] Zuo J, Dowell AC, Pearce H, Verma K, Long HM, Begum J, et al. Robust SARS-CoV-2-specific T cell immunity is maintained at 6 months following primary infection. Nat Immunol. 2021;22(5):620–6. doi: 10.1038/s41590-021-00902-8 33674800 PMC7610739

[97] Sekine T, Perez-Potti A, Rivera-Ballesteros O, Strålin K, Gorin J-B, Olsson A, et al. Robust T Cell Immunity in Convalescent Individuals with Asymptomatic or Mild COVID-19. Cell. 2020;183(1):158-168.e14. doi: 10.1016/j.cell.2020.08.017 32979941 PMC7427556

[98] Gallais F, Velay A, Nazon C, Wendling M-J, Partisani M, Sibilia J, et al. Intrafamilial Exposure to SARS-CoV-2 Associated with Cellular Immune Response without Seroconversion, France. Emerging Infectious Diseases. 2021;27(1):113–21. 33261718 10.3201/eid2701.203611 PMC7774579

[99] Al-Sheboul SA, Brown B, Shboul Y, Fricke I, Imarogbe C, Alzoubi KH. An Immunological Review of SARS-CoV-2 Infection and Vaccine Serology: Innate and Adaptive Responses to mRNA, Adenovirus, Inactivated and Protein Subunit Vaccines. Vaccines (Basel). 2022;11(1):51. doi: 10.3390/vaccines11010051 36679897 PMC9865970

[100] Paniskaki K, Konik MJ, Anft M, Heidecke H, Meister TL, Pfaender S, et al. Low avidity circulating SARS-CoV-2 reactive CD8 T cells with proinflammatory TEMRA phenotype are associated with post-acute sequelae of COVID-19. Frontiers in Microbiology. 2023;14. 10.3389/fmicb.2023.1196721 PMC10272838 37333646

[101] Neidleman J, Luo X, Frouard J, Xie G, Gill G, Stein ES, et al. SARS-CoV-2-Specific T Cells Exhibit Phenotypic Features of Helper Function, Lack of Terminal Differentiation, and High Proliferation Potential. Cell Rep Med. 2020;1(6):100081. doi: 10.1016/j.xcrm.2020.100081 32839763 PMC7437502

[102] Neidleman J, Luo X, George AF, McGregor M, Yang J, Yun C, et al. Distinctive features of SARS-CoV-2-specific T cells predict recovery from severe COVID-19. Cell Rep. 2021;36(3):109414. doi: 10.1016/j.celrep.2021.109414 34260965 PMC8238659

[103] Mele D, Ottolini S, Lombardi A, Conteianni D, Bandera A, Oliviero B, et al. Long‐term dynamics of natural killer cells in response to SARS‐CoV‐2 vaccination: Persistently enhanced activity postvaccination. Journal of Medical Virology. 2024;96(4). 10.1002/jmv.29585 38566585

[104] Maucourant C, Filipovic I, Ponzetta A, Aleman S, Cornillet M, Hertwig L, et al. Natural killer cell immunotypes related to COVID-19 disease severity. Sci Immunol. 2020;5(50):eabd6832. doi: 10.1126/sciimmunol.abd6832 32826343 PMC7665314

