# Supplementary figures and images for: Detectable SARS-CoV-2 specific immune responses in recovered unvaccinated individuals 250 days post wild type infection
Source: PLoS One. 2025 Jun 11;20(6):e0325923. doi: 10.1371/journal.pone.0325923 (PMC12157120; doi:10.1371/journal.pone.0325923)

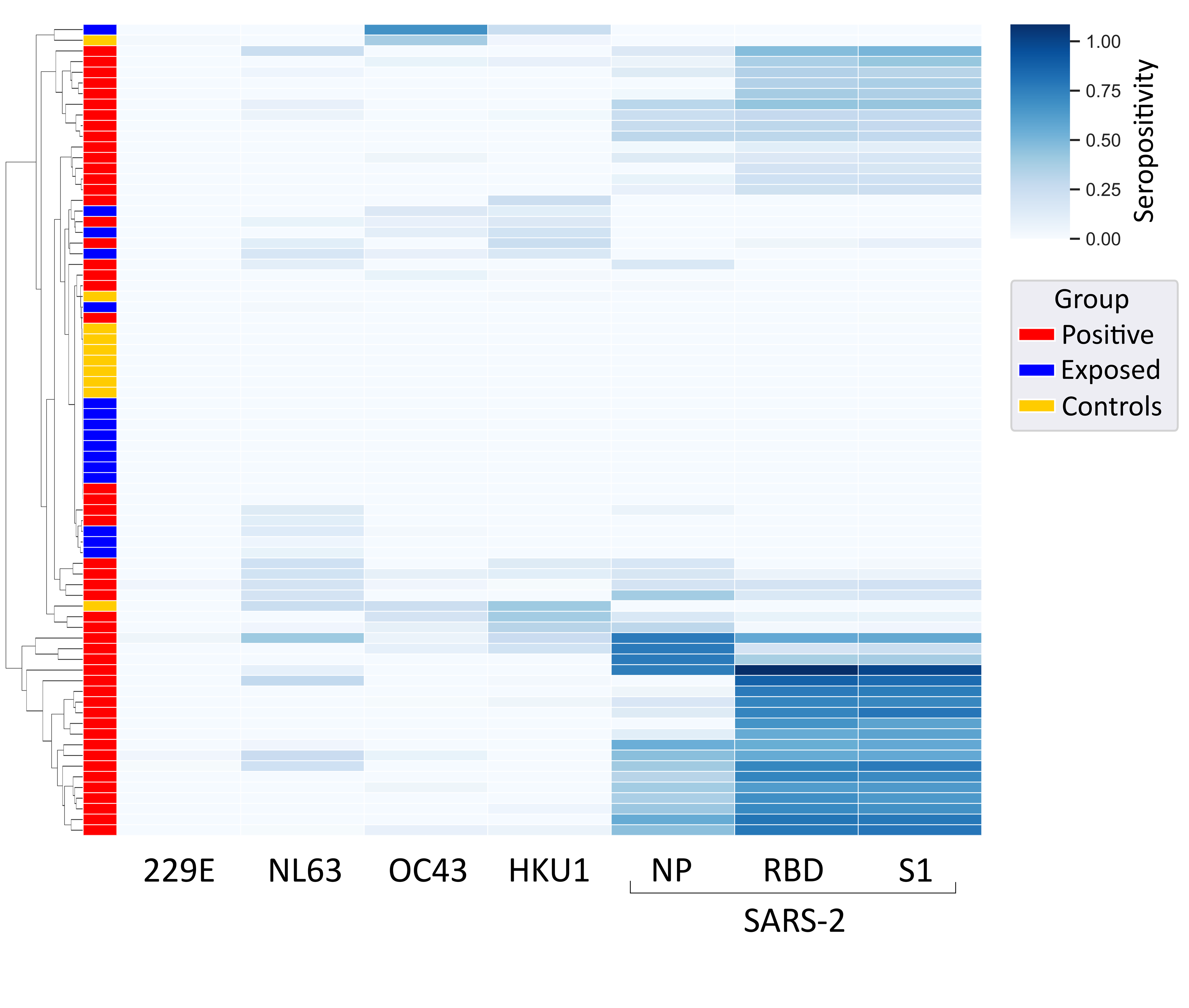

Supplement: S1 Fig — Shown are the results of the serological tests for SARS-CoV2 and HCoVs clustered by the strength of response (seropositivity) in the ICS subset. The exposed (blue) and control (yellow) groups did – by definition – not show a signally serological answer against SARS-CoV-2. The positive (red) group prominently features the bottom right cluster of samples that had a strong serological answer against SARS-CoV-2. This cluster is subdivided into smaller clusters separated by their answer to the different SARS-CoV-2-antigens. (TIF) [file pone.0325923.s001.tif]

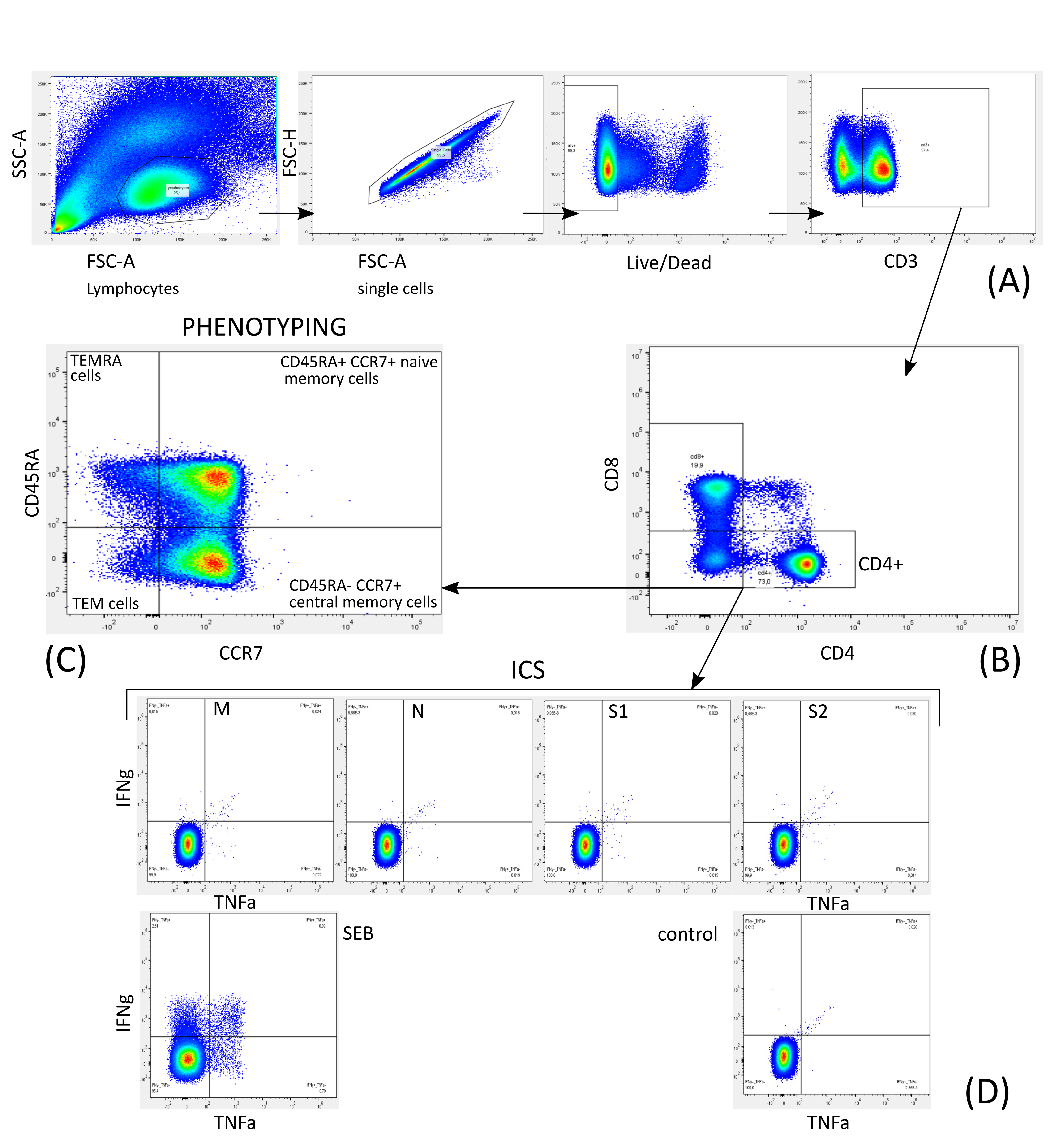

Supplement: S2 Fig — Example of the FACS-gating strategy used to determine the extents of the T cell populations. Shown is the gating procedure, used to determine subpopulations of the PBMC population (lymphocytes - > single cells - > alive cells - > CD3+ cells) (A). Subfigure (B) shows the differentiation for CD4+ T cells. Subfigure (C) shows the differentiation between CCR7 and CD45RA CD4+ T cell populations used in the phenotyping protocol. Subfigures (D) show the IFNγ and TNFα producing CD4+ T cell subsets. (D) depicts results of stimulation with SARS-CoV-2 peptides M, N, S1 and S2 as well as the stimulation controls (SEB and control). (TIF) [file pone.0325923.s002.tif]

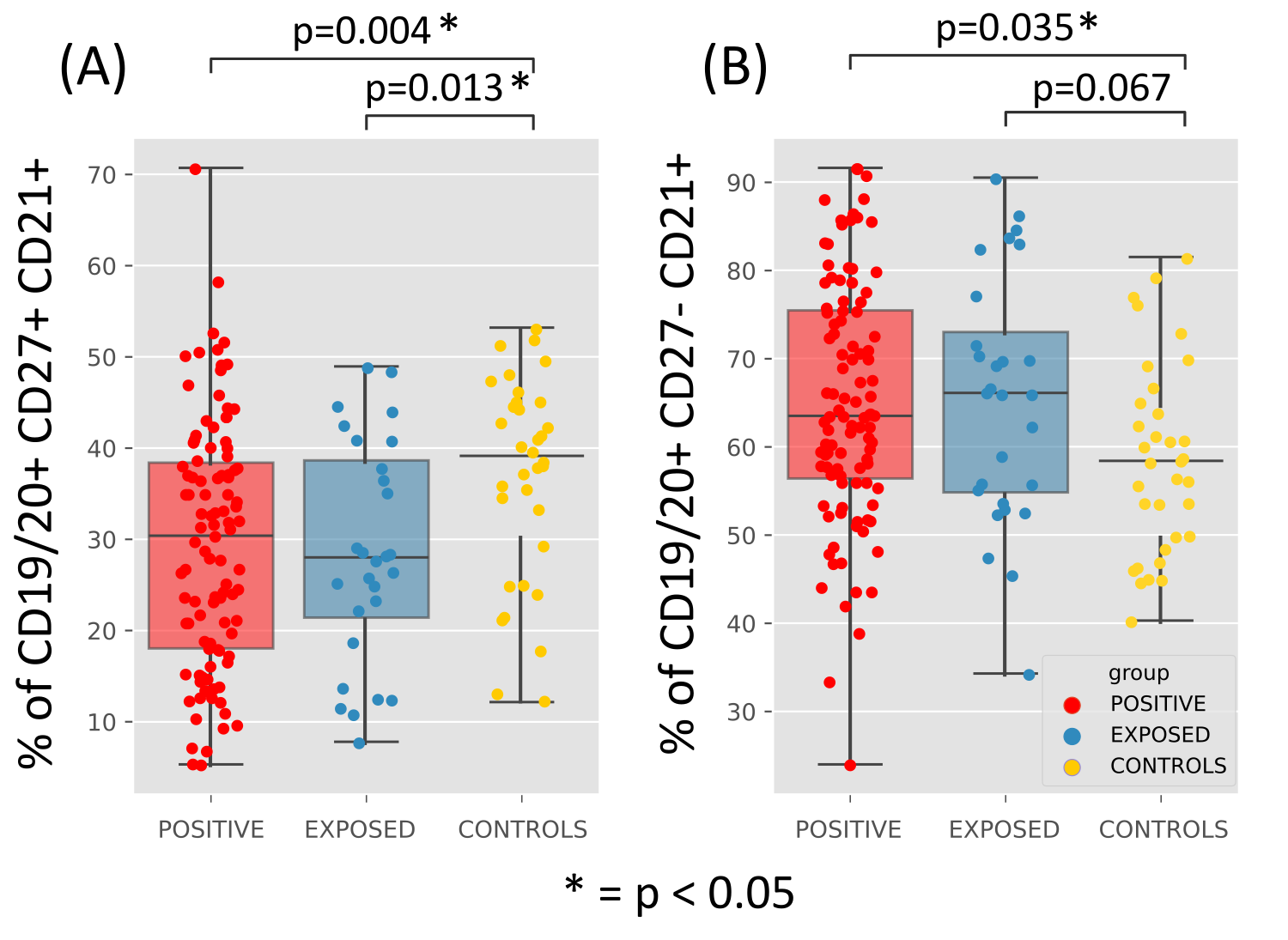

Supplement: S3 Fig — Shown are CD27+ CD21+ (A) and CD27- CD21+ (B) subsets of mature (CD19+ /CD20+) B cells in comparison between the positive (red), exposed (blue) and control group (yellow). Statistics were performed using the Kruskal-Wallis-test and Dunne’s post hoc test and adjusted using the Benjamini-Hochberg-correction. (TIF) [file pone.0325923.s003.tif]
